# Effect of Long-Term Hydroxytyrosol Administration on Body Weight, Fat Mass and Urine Metabolomics: A Randomized Double-Blind Prospective Human Study

**DOI:** 10.3390/nu14071525

**Published:** 2022-04-06

**Authors:** Christina Fytili, Theodora Nikou, Nikolaos Tentolouris, Ioulia K. Tseti, Charilaos Dimosthenopoulos, Petros P. Sfikakis, Dimitrios Simos, Alexandros Kokkinos, Alexios L. Skaltsounis, Nikolaos Katsilambros, Maria Halabalaki

**Affiliations:** 1 First Department of Propaedeutic and Internal Medicine, Medical School, National and Kapodistrian University of Athens, Laiko General Hospital, 17 Agiou Thoma Street, 11527 Athens, Greece; ntentol@med.uoa.gr (N.T.); psfikakis@med.uoa.gr (P.P.S.); simosd@gmail.com (D.S.); rjd@otenet.gr (A.K.); 2 Department of Pharmacy, Division of Pharmacognosy and Natural Products Chemistry, National and Kapodistrian University of Athens, Panepistimioupoli Zografou, 11527 Athens, Greece; th-nikou@pharm.uoa.gr (T.N.); skaltsounis@pharm.uoa.gr (A.L.S.); 3 Uni-Pharma SA, 14th klm National Road 1, Kifissia, 14564 Athens, Greece; jtsetis@uni-pharma.gr; 4 Department of Clinical Nutrition, Laiko General Hospital, 17 Agiou Thoma Street, 11527 Athens, Greece; harisdimos@gmail.com; 5 Research Laboratory Christeas Hall, Medical School, National and Kapodistrian University of Athens, 15b Agiou Thoma Street, 11527 Athens, Greece; nicholaskatsilambros@gmail.com

**Keywords:** hydroxytyrosol, human cohort, obesity, weight and fat loss, UPLC-Orbitrap-MS, metabolomics

## Abstract

Hydroxytyrosol (HT) is a natural antioxidant found in olive products and characterized by well-documented beneficial effects on human health. Several research studies are ongoing that aim to investigate its potency and molecular mechanism of action. The present study aimed to investigate the potential effect of HT on human obesity through a randomized double-blind prospective design. HT in two different doses (15 and 5 mg/day) and a placebo capsule was administered to 29 women with overweight/obesity for six months and their weight and fat mass were monitored at three time points (baseline, 4, 12 and 24 weeks). Statistically significant weight and visceral fat mass loss (%weight loss: *p* = 0.012, %visceral fat loss: *p* = 0.006) were observed in the group receiving the maximum HT dosage versus placebo after 4 weeks of the intervention, with attenuation of these findings at 12 and 24 weeks of the study. Urine samples were collected during the intervention and analyzed via liquid chromatography–high-resolution mass spectrometry for untargeted metabolomic purposes and comparisons between study groups were performed. HT administration was safe and well-tolerated. To the best of our knowledge, this is the first human cohort investigating the effects of HT on obesity for a prolonged study period.

## 1. Introduction

Obesity is considered a major public health concern and based on the World Health Organization’s most recent data, it affects more than 39% of the world’s population [1]. Overweight and obesity are defined as abnormal or excessive fat accumulation that may impair health and are generally measured using the body mass index (BMI) [1]. BMI is determined as weight in kilograms (kg) divided by the square of height in meters (kg/m^2^). Overweight is defined as a BMI ranging from 25–29.9 kg/m^2^ and obesity as a BMI > 30 kg/m^2^ for people of Caucasian origin [2]. Obesity is usually accompanied by chronic low-grade inflammation and other metabolic disorders, such as type 2 diabetes, disorders of lipid and glucose homeostasis, cardiovascular diseases and hepatic steatosis [3].

Obesity is induced both by genetic and lifestyle factors. Generally, it is caused by the imbalance between caloric intake and expenditure, usually due to the consumption of energy-dense foods and lack of physical activity [4]. Lately, a growing number of studies have focused on the gut microbiome and specifically on changes in symbiotic bacteria populations and equilibria in the gastrointestinal tract and the impact of such changes on human metabolic disorders [5]. The gut microbiome has a recognized contribution to digestion and metabolism by regulating energy production and fatty acid tissue composition. Investigation of the gut microbiome population and its effects on human health are nowadays the center of scientific interest [6].

These two determinant factors, namely, caloric intake and microbiota composition, are affected by ethnic and individual dietary habits. The Mediterranean diet (MD) is considered one of the healthiest diets and its cardioprotective effect is well documented. Higher adherence to the MD is associated with lower abdominal adiposity in Mediterranean populations [7,8], prevention of obesity, metabolic syndrome and its related disorders [9]. Olive products are major components of the MD, containing a plethora of phenol and secoiridoid derivatives, which are known for their antioxidant and anti-inflammatory properties [10]. One of these compounds is hydroxytyrosol (3,4-dihydroxyphenylethanol, HT), which is a bioactive phenylethanol with a catechol moiety in olive products, with confirmed substantial antioxidant, anti-inflammatory and antimicrobial properties [11]. Likewise, HT consumption is related to the amelioration of metabolic syndrome and related disorders [12], while it was recently found that it improves obesity and insulin resistance by modulating gut microbiota [3]. In addition, HT was found to prevent inflammation and hyperglycemia produced by a high-fat diet [13,14] and decrease liver steatosis [14].

Additionally, HT is a characteristic phenol of olive oil (OO) and is strongly associated with the protection of blood lipids from oxidative stress. After a European Food Safety Authority (EFSA) investigation, the above positive effect was recognized, suggesting a daily consumption of OO containing at least 5 mg of HT and its derivatives per 20 g of OO [15]. HT also shows high concentrations in olive drupes and leaves [16,17] and is also present in red wine [18] and several other species of the Oleaceae family [19]. HT can also be found in considerably high amounts in the OO production by-products. Additionally, another rich source of HT is the water from the debittering process of edible olives. Its concentration in all these products depends on several factors, such as the olive cultivar, production procedure parameters, agronomic practices and storage conditions [16]. It should also be mentioned that HT is normally synthesized in the human body, where it is formed through the dopamine metabolic pathway [20]. HT and the intermediate metabolites of the dopamine pathway, namely, homovanillic alcohol, 3,4-dihydroxyphenylacetic acid (DOPAC) and homovanillic acid, are physiologically present in biological matrices in variable concentrations and they are typically considered as dopamine biomarkers, along with HT [20].

Regarding bioavailability, HT is mainly absorbed in the intestine through a bi-directional passive diffusion mechanism with an efficiency ranging from 75% up to 100% [21]. The absorption is affected by the matrix and formulation of the HT vehicle of administration, with it being higher as a constituent of OO than in its pure form [22]. In vivo studies have shown that sex is also a critical factor for HT bioavailability, which persists for a longer time in female versus male rats [23]. HT shows an intense and rapid absorption rate, and its plasma half-life is around 1–2 min. Once absorbed, it is strongly bound to high-density lipoproteins acting as an antioxidant and cardioprotective factor when administered intravenously [24]. HT and its metabolites are highly distributed to tissues such as muscle, testis, liver and brain (HT is able to cross the blood–brain barrier) and are generally accumulated in the kidneys and liver [25].

Only a few studies have been conducted to investigate the effects of OO biophenols and, more specifically, HT in obesity. HT increases the expression of genes involved in the inhibition of adipogenesis, while it downregulates the expression of genes involved in promoting adipogenesis in human visceral adipocytes [26]. In animal studies, treatment of high-fat diet-induced diabetic mice with high doses of HT (20 mg/kg/day orally for 3 weeks) resulted in reduced weight gain and visceral fat deposits [27], though conflicting results were found from other animal studies using smaller doses of HT (4 mg/kg [28], 3 mg/kg [29], 8 and 16 mg/kg body weight [30]).

Data from human studies in this area are limited and conflicting. In a human volunteer study of 14 people with mild hyperlipidemia, the daily administration of an aqueous solution containing 45 mg of HT for 8 weeks along with dietetic consultation (but not caloric restriction) did not result in significant changes in body weight [31]. Likewise, no changes in body weight were noticed after the administration of 51.1 mg of oleuropein and 9.7 mg HT in male volunteers for 12 weeks, despite an observed 15% increase in insulin sensitivity [32].

However, in a crossover study of 11 patients with overweight and type 2 diabetes (T2DM), the daily consumption of extra virgin OO enriched with 14.425 mg of phenols (though with an unspecified HT concentration) versus placebo for 4 weeks without diet modifications, significantly reduced BMI, body weight, fasting plasma glucose, glycated hemoglobin and visfatin levels [33]. Similarly, statistically significant body weight and fat mass reductions were demonstrated after daily consumption of 15 mg of HT in the form of gastroresistant capsules for 3 weeks without dietetic restriction [34].

Based on the above data, conclusions regarding the effect of HT on the metabolic profile and obesity cannot be safely drawn. This may be attributed to the small number of studies; the variability in study design and duration; and the variation between study groups (i.e., age, sex, ethnicity, baseline metabolic status), posology and form of HT used [35].

HT is easily detected in human urine in its free form and several hyphenated techniques, such as gas chromatography–mass spectrometry (GC-MS) and liquid chromatography–mass spectrometry (LC-MS), were proposed for quantification purposes [36]. It was proposed that HT absorption and excretion levels are positively correlated with the administered doses [37]. In contrast, HT detection in human plasma is rather arduous due to its high binding affinity to plasma lipoproteins, which makes it almost undetectable in human plasma in its free form. For this reason, most ADMET (absorption, distribution, metabolism, elimination, toxicity) studies propose alternative HT biomarkers, usually conjugated forms, for the investigation of HT absorption and metabolism [38]. Usually, several time-consuming steps are required in the sample preparation process, including derivatization reactions and expensive purification steps [39]. In parallel, low analyte recovery hampers plasma studies. Consequently, urine is regarded as the most suitable biological fluid for the investigation of HT metabolism.

In the present work, a randomized double-blind intervention study was designed to investigate the effect of HT in women with overweight and obesity, in combination with diet and physical exercise. For this purpose, an encapsulated extract standardized in HT was developed and administered to participants in two different doses (5 and 15 mg/day) for six months and the results were compared to those from participants receiving placebo capsules. The HT extract was derived from edible olive debittering water, with a final concentration of 2.5 mg HT per capsule. Urine samples were collected at three time points (baseline: T0, 3 months: T3 and 6 months: T6) during the intervention period. An ultra-performance liquid chromatography-high resolution mass spectrometry (UPLC-HRMS) platform was employed for untargeted metabolomic analysis of urine samples with the aim to explore HT metabolism and disclose possible biomarkers.

## 2. Materials and Methods

### 2.1. Chemicals and Reagents

The acetonitrile (ACN), *n*-hexane and hydrochloric acid (HCl) used for the HT capsule pretreatment were of analytical grade (Fisher, Hampton, VA, USA). The ACN, water (H_2_O) and acetic acid used for the HT quantification in capsules were of HPLC grade (Fischer, Hampton, VA, USA). The ACN and formic acid used for HRMS analysis were LC-MS grade (Fisher, Hampton, VA, USA) and H_2_O was obtained from a Milli-Q water purification system (Millipore, Burlington, MA, USA).

### 2.2. Instrumentation

Quantification of HT in capsules was achieved using a high-performance liquid chromatography (HPLC) system equipped with a pump SpectraSystem P4000, autosampler SpectraSystem AS3000 and PDA SpectraSystem UV800. Metabolomic analysis of urine was performed using an H-class Acquity UPLC system (Waters, Milford, MA, USA) coupled with an LTQ-Orbitrap XL hybrid mass spectrometer (Thermo Scientific, Waltham, MA, USA).

### 2.3. Capsules Quantitative and Qualitative Analysis

Soft capsules were developed for the conduction of the human cohort, HT and placebo. In both capsules, refined OO was used as a carrier with the complete absence of biophenols. The HT capsules were enriched in HT that originated from edible olive debittering water as described by Xynos et al. [40] and standardized to a final concentration of 2.5 mg/capsule.

For the quantification of HT in capsules, special treatment was employed. The capsule outer cover was deconstructed with incubation for 90 min at 37 °C with adjusted pH = 2 using HCl. After incubation, the solution was filtered with a 0.22 µm PVDF filter and then directly analyzed via HPLC-DAD (more information in Supplementary Materials). For the identification of compounds in HT and placebo capsules, UPLC-Orbitrap-MS analysis was employed (more information in Supplementary Materials). The capsules were incised and the outer cover was separated from the inner content. The capsule content was diluted in ACN and *n*-hexane in a 1:1 ratio for defatting, using liquid–liquid extraction. The organic phase was evaporated to dryness. Prior to analysis, the samples were reconstituted with H_2_O:MeOH/80:20 to a final concentration of 200 μg/mL. Mass spectra were obtained in negative and positive ionization and were recorded in full scan mode in the range of 115–1000 *m*/*z*, with a resolving power of 30,000 at 500 *m*/*z* and a scan rate of 1 microscan per second. HRMS/MS experiments were obtained using a data-dependent method with a collision energy of 35.0% (q = 0.25). Analysis was carried out for both types of capsules for comparison purposes.

### 2.4. Hydroxytyrosol Capsule Administration to Women with Overweight/Obesity

#### 2.4.1. Study Design

The study was designed to be prospective, randomized, double-blinded and placebo-controlled with a total duration of 6 months for each participant. Enrollment was gradually performed and the study was conducted between October 2017 and May 2019. Participants were recruited from the Obesity Outpatient Clinic of the Diabetes Centre of the First Department of Propaedeutic and Internal Medicine, Medical School of the National and Kapodistrian University of Athens (NKUA), Laiko General Hospital. All visits and measurements were performed in the “Nikolaos L Katsilambros” Laboratory of the Diabetes Centre of the same institution. The study protocol was reviewed and approved by the Ethics Committee of the Laiko General Hospital, Athens, Greece, and is registered with ClinicalTrials.gov (NCT: 04317079). All participants provided written informed consent before recruitment in accordance with the provisions of the Ethics Committee and with the Helsinki Declaration of 1975, as revised in 1983.

#### 2.4.2. Sample Size Analysis

An a priori sample size analysis was made. The sample size was calculated to provide a statistical power of 95% and an α error probability of 0.05. We assumed that the placebo group would have a weight loss of 4 kg in the first four weeks of the study and the intervention group would have a weight loss of 6 kg with a standard deviation (SD) of 1 kg; based on these, 21 participants should be enrolled. Calculating an increase of 12% of the sample size to achieve statistical power due to a 2:1 randomization rate of the study design and a dropout rate of 20%, the minimum sample size was defined as 29.

#### 2.4.3. Sample Characteristics and Study Protocol

Thirty-seven otherwise healthy women with overweight or obesity with BMIs of 26.64–35.68 kg/m^2^ and a stable body weight (defined as less than 5% variation) for three months prior to enrollment were randomized in a double-blind intervention design. The participants were aged 22–66 years old (mean age of 48.55 years). Major health problems, diabetes mellitus, pregnancy, lactation and administration of weight-modifying medications were exclusion criteria.

The selection of female subjects was based on literature data showing that HT has enhanced absorption in female rats [23].

Participants were randomized in a 2:1 ratio into intervention and placebo groups. The intervention group received HT and was divided based on baseline body fat mass percentage into two groups: group A received 15 mg of HT daily if their body fat mass percentage was >40% at baseline and group B received 5 mg of HT daily if their body fat mass percentage at baseline was <40%. Group C (placebo) received placebo capsules (Table 1).

The dose of 5 mg, which is the daily consumption of HT suggested by the EFSA with documented benefits on lipid profile [15], was chosen in order to investigate potentially additional benefits of this posology on body weight and composition. However, as HT properties are known to be dose-dependent [41], we assumed that larger doses may be needed, especially in participants with more severe obesity, to demonstrate possible effects. Favorable effects were shown in short-term studies with the dose of 15 mg [34]; therefore, our study included a second intervention arm for patients with more severe obesity using the daily dose of 15 mg. The randomization was based on fat mass percentage and not BMI, as the latter does not directly reflect body composition.

**Table 1:** Description of intervention groups. Each hydroxytyrosol (HT) capsule contained 2.5 mg of HT. For each group, the total HT intake and capsule consumption per day is illustrated. In the last row, the number of volunteers of each group is presented.

Group	Group A	Group B	Group C
HT Intake	15 mg HT/Day	5 mg HT/Day	0 mg/Day
Capsule consumption (per day)	6 HT capsules (2 HT capsules before three main meals)	2 HT and 4 placebo capsules (1 HT and 1 placebo capsule before breakfast, 2 placebo capsules before lunch, 1 HT and 1 placebo capsule before dinner)	6 placebo capsules (2 placebo capsules before three main meals)
Number of volunteers	9	9	11

Participants were advised to consume the capsules 30 min before main meals in all circumstances, whereas in the cases of simultaneous administration of other medications, a 2 h time interval between the administration of HT and other substances was advised.

At the baseline visit (T0), the medical history was recorded and anthropometric measurements were carried out: weight and height for the BMI estimation and determination of body and visceral fat. Body weight, body fat mass and body fat mass percentage were calculated using bioelectrical impedance analysis via Tanita Body Composition Analyzer BC-418, while visceral fat mass was calculated using Tanita ViScan AB-140 equipment (Tanita Corporation Ltd., Tokyo, Japan). All measurements were performed at least twice in order to ensure repeatability. Subsequent visits were performed at 1 (T1), 3 (T3) and 6 months (T6) of the intervention. During each visit, all measurements were repeated, while urine samples were collected at baseline T0, T3 and T6.

All participants and investigators were blinded to the group assignments. Before initiation of the trial, HT and placebo capsules were prepackaged by an independent investigator with no subsequent involvement in the study and coded with random non-continuous numbers: one group concerning participants with a fat mass over 40% of total body weight included packages with either 15 mg HT daily or placebo and the other group concerning participants with fat mass percentage less than 40% of body weight included capsules either containing 5 mg HT daily or placebo. Based on the fat mass percentage measurement at the baseline visit, a random code number was given to each participant and the randomization was revealed after the completion of the intervention.

During the 6 months of the intervention, participants were consulted at each visit by a dietitian and followed a personalized hypocaloric diet based on their estimated basal metabolic rate, following the principles of the MD in all cases. During each visit, adherence to the diet was assessed with the use of diet-recalling questionnaires, the level of physical activity was recorded and compliance with capsule consumption was evaluated by checking the empty blister packs.

From the initial list of participants, 3 women withdrew from the study due to an inability to attend the program, and data from 5 other women, despite completing the intervention, were not included in the data analysis due to inadequate compliance with the diet and HT consumption. Hence, data from 29 women were used in the statistical analysis.

#### 2.4.4. Studied Anthropometric Parameters and Statistical Analysis

Body weight, fat mass and visceral fat were recorded at T1, T3 and T6 and compared to the baseline. Statistical analysis was performed using SPSS (Statistical Package for Social Sciences, IBM corporation, Version 21.0, Armonk, NY, USA) for data from the 29 participants that complied with the diet, exercise and capsule consumption regimens. The Skewness and kurtosis normality of residuals test and the Shapiro–Wilk test were employed to determine the normality of data distribution at baseline. Variables with normal distribution were analyzed utilizing the *t*-test for independent samples. When not satisfying normality, the Mann–Whitney test was employed. A *p*-value < 0.05 was considered the significant statistical value in order to assess differences between intervention groups throughout the study.

Univariate analysis of covariance (ANCOVA) was further performed to examine whether the observed variations of body weight, fat mass and visceral fat differed between the intervention and placebo groups while controlling for the baseline value of each parameter. The same analysis was repeated for comparisons between groups A and C as well.

#### 2.4.5. Urine Collection and Sample Preparation

Urine samples for untargeted metabolomics were collected in sterile urine collection vessels and stored at −80 °C until subsequent analysis. Initially, samples were thawed in ice, homogenized and then 1.5 mL was centrifuged at 12,000 rpm for 10 min at 4 °C. A total of 50 μL of each sample code were added in an Eppendorf tube. Subsequently, 200 μL of cold methanol (MeOH) were added and homogenized with a vortex for 60 s. Finally, samples were centrifuged at 4 °C for 12 min at 14,000 rpm and supernatants were evaporated under vacuum conditions and centrifugation at room temperature until the pellets were completely dry. The derived residues were diluted in 100 μL MeOH:H_2_O/60:40. Pooled samples were also prepared and used as quality control (QC) samples. All samples were analyzed in triplicate with the developed UPLC-Orbitrap MS method described below.

### 2.5. Untargeted Metabolomics Analysis of Urine Samples

#### 2.5.1. Samples

In total, 63 urine samples collected from the participants at three time points (T0, T3, T6) were forwarded for metabolomic analysis. Table S1 in the Supplementary Materials illustrates the analyzed samples in detail.

#### 2.5.2. UPLC-HRMS Analysis

Urine extracts, as well as the QC pooled sample, were analyzed with an LC gradient consisting of H_2_O with 0.1% formic acid (solvent A) and ACN (solvent B). The elution method started with 2% of B, which stayed for 2 min. In the next 16 min, B reached 100% and stayed for 2 min. Finally, at the 21st min, the system returned to the initial conditions and stayed for 4 min for system equilibration. A Thermo Hypersil Gold C-18 (50 mm × 2.1 mm, 1.9 μm) column was used for the separation, with a stable temperature of 40 °C. The measurements were performed with a total acquisition time of 25 min and a flow rate of 400 μL/min. The injection volume was 10 μL and the autosampler temperature was 7 °C.

High-resolution mass spectra (HRMS) were obtained in negative and positive ion modes using an electrospray ionization source (ESI). The capillary temperature was set at 350 °C and the sheath and auxiliary gases were adjusted to 40 and 10 arb, respectively. For the negative ionization the capillary temperature was set to 350 °C, the capillary voltage at −30 V and the tube lens at −100 V. For the positive ionization, only the capillary voltage and the tube lens were adjusted to 40 V and 120 V, respectively. Mass spectra were recorded in full scan mode in the range of 115–1000 *m*/*z*, with a resolving power of 30,000 at 500 *m*/*z* and a scan rate of 1 microscan per second. HRMS/MS experiments were obtained using a data-dependent method with a collision energy of 35.0% (q = 0.25). The system was calibrated externally every 50 injections (details of the acquisition in Supplementary Materials).

#### 2.5.3. Statistical Process and Chemometrics

UPLC-HRMS acquisitions were recorded with Xcalibur 2.2. Raw files (.raw, Thermo) were imported into MZmine 2.26 software for data processing. Peak lists were generated with a centroid selection algorithm. For chromatogram building of the generated mass lists, 0.03 min was set as the minimum time of span and 5 ppm for the mass tolerance. A chromatogram deconvolution module was employed, and spectra were processed with a local minimum search algorithm using the R package. The minimum retention time range was set to 0.03 min and a peak width of 0.05–0.5 min. Chromatograms were aligned and spectra were normalized regarding the retention time with a 0.05 min tolerance. The join alignment calculation method based on the mass and detection time of each peak was used. Finally, gap filing was implemented, using a peak-finder method. This list was exported as a CSV file and imported into SIMCA 14.1 (Umetrics, version, city, Sweden) software for statistical analysis. Mainly, principal component analysis (PCA) and orthogonal partial least squares (OPLS) methods were implemented. The generated models were evaluated for their R2 and Q2 parameters indicating the measure of fit and the predictability, respectively. Only models with R2 values close to 1, Q2 values over 0.5 or models with lower R2 but close to the Q2 value were accepted. A permutation test was also applied for further validation of the models (Figure S12 in Supplementary Materials).

## 3. Results and Discussion

### 3.1. Quantitative and Qualitative Capsule Analysis

As mentioned before, HT is a characteristic compound of olive products and by-products as well. Therefore, debittering water (DW), which is produced during the processing of table olives, was used for capsule preparation due to its high content in HT [40]. Briefly, DW coming from table olives of the Amfissis variety (10.10% *w*/*w* richness of HT) was extracted with XAD-4 resins, as described by Xynos and co-workers [40]. The produced DW extract was then encapsulated with a soft outer cover. Refined OO was used as a carrier in both types of capsules, i.e., HT and placebo, based on previous studies showing that HT is more effectively absorbed in humans when administered in an OO matrix [22]. The two types of capsules looked identical to protect the double blindness of the study.

**Figure 1:**
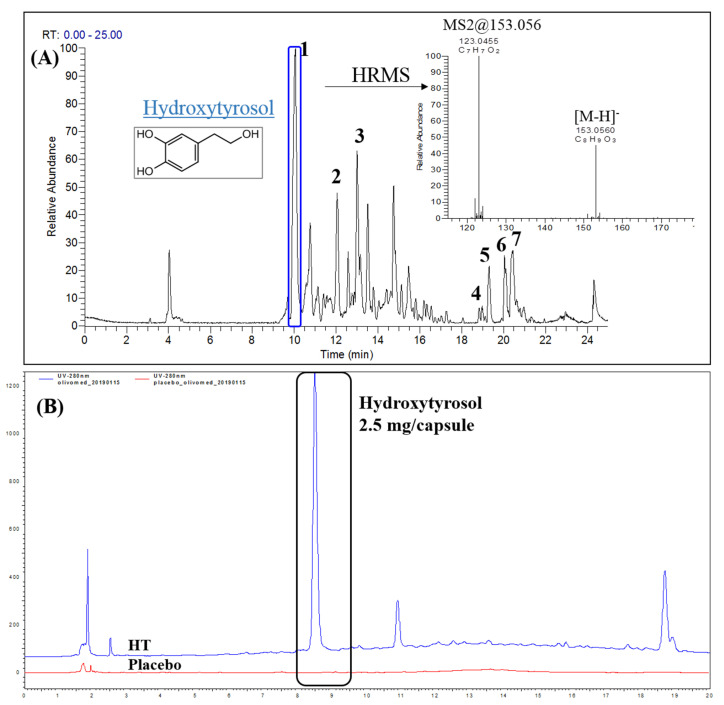
(**A**) Base peak (BP) chromatogram of the hydroxytyrosol (HT) capsule extract. Annotated peaks 1–7 correspond to the identified metabolites in the extract. On the right side of the chromatogram, the high-resolution mass spectrometry (HRMS) spectrum of HT is presented. [M-H]^-^ corresponds to the pseudomolecular ion of HT in negative ionization and its characteristic on-source fragment (*m*/*z* 123.0455). (**B**) High-performance liquid chromatography–diode-array detection (HPLC-DAD) chromatogram at 280 nm of hydroxytyrosol (HT) capsule (blue) and placebo capsules (red) after dialysis in stomach conditions. The HT peak is highlighted.

After the production of the capsules, both types (HT and placebo) were subjected to UPLC-Orbitrap-MS analysis to ensure the absence of HT from the placebo. Another objective of this analysis was to explore the presence of additional compounds in HT capsules that could possibly release HT and cumulatively contribute to its total concentration level. Indeed, in the placebo capsules, HT was not detected, even in traces, as shown in the base peak chromatograms in Figure S1 of the Supplementary Materials. Furthermore, as initially hypothesized, other compounds were also detected in HT capsules, as observed in Figure 1A. Therefore, taking advantage of the high resolving power and high accuracy of the Orbitrap analyzer, in combination with the separation of the UPLC dimension, we tried to identify these compounds with an emphasis on possible derivatives of HT. A molecular formula (MF) and ring and double bong equivalence (RDBeq) in parallel with the observed isotopic patterns and the HRMS/MS spectra contributed to the identification procedure. The negative ion mode revealed better ionization of compounds and was ultimately used for the identification process. Generally, agents from the encapsulation process and fatty acids (FA) from the carrier were identified together with HT and extract constituents. In total, seven metabolites were identified (Table 2), while unknown peaks are also given in Supplementary Materials (Table S2).

In detail, in the HT capsule, HT (**1**) was identified at *m*/*z* 153.0561, as expected. Additionally, the dialdehydic form of decarboxymethyl elenolic acid (**2**) *(m*/*z*: 183.0665), HT acetate (**3**) (*m*/*z*: 195.0656), octadecanedioic acid (**4**) (*m*/*z*: 313.2379), linoleic acid (**5**) (*m*/*z*: 279.2325), palmitic acid (**6**) (*m*/*z*: 255.2328) and oleic acid (**7**) (*m*/*z*: 281.2483) were also detected. The identified metabolites **1**–**7** presented in Table 2 are annotated in Figure 1A and their respective HRMS spectra are shown in Figures S2–S8 in the Supplementary Materials. The identified metabolites **1**–**3** are typical compounds of the enriched HT extract and metabolites **4**–**7** are the characteristic FA of OO, which was used as the carrier of the extract during the encapsulation process. For this reason, the placebo capsule was composed only of metabolites **4**–**7** and did not contain biophenols at all. It has to be underlined that both capsules contained the same OO as carrier and, as a result, the two capsules had the same FA composition. As already mentioned, oleic acid was reported as a satiety factor [42]. The use of the same OO in capsules reassured us that the obtained results of the study were caused by HT and not by the contained FA, which were received by all the groups of the study.

**Table 2:** Identified compounds in the extract of the hydroxytyrosol (HT) capsule. The identification parameters are presented in each column. *m*/*z* exp: experimental *m*/*z*; *m*/*z* theor: theoretical *m*/*z*; RT: retention time; MF: molecular formula; RDBeq.: ring and double bond equivalence; *δ*: the mass error measured in ppm.

	*m*/*z* Exp	*m*/*z* Theor	RT (min)	MF	RDBeq.	*δ* (ppm)	Compound
**1**	153.0561	153.0546	10.05	C_8_H_10_O_3_	4.5	1.650	Hydroxytyrosol
**2**	183.0665	183.0652	12.06	C_9_H_12_O_4_	4.5	0.917	Dialdehydic form of decarboxymethyl elenolic acid
**3**	195.0656	195.0652	13.19	C_10_H_12_O_4_	5.5	0.963	Hydroxytyrosol acetate
**4**	313.2379	313.2373	18.82	C_18_H_34_O_4_	2.5	−1.861	Octadecanedioic acid
**5**	279.2325	279.2319	20.09	C_18_H_32_O_2_	3.5	−1.588	Linoleic acid
**6**	255.2328	255.2319	21.45	C_16_H_34_O_2_	1.5	−0.719	Palmitic acid
**7**	281.2483	281.2475	21.60	C_18_H_34_O_2_	2.5	−1.222	Oleic acid

Based on the LC-MS data results, our initial hypothesis of other compounds being present in the extract that could affect the HT concentration was verified. Thus, to quantify the HT in the capsules, a specific protocol was used in acidic conditions. In these conditions, the hydrolysis of such compounds, e.g., hydroxytyrosol acetate, was achieved. Therefore, the concentration of HT could be accurately determined according to the EFSA suggestion, which was used to design the dosing scheme of the study. On the other hand, these conditions (pH = 2 and T = 37 °C) simulated human gastric conditions after oral consumption. It is important to note that HT is stable under these conditions [43]. The protocol was applied to both the HT and placebo capsules. The quantification was based on an eight-point calibration curve constructed using the area of HT at 280 nm. The equation was y = 87,793x − 35,339 with R^2^ = 0.99998. The HT concentration was estimated at 2.47 mg HT per capsule. Figure 1B depicts the HPLC-DAD chromatogram of the acidic treatment of HT and placebo capsules acquired at 280 nm. As was obvious, placebo capsules were completely free of HT.

### 3.2. Effect of the Intervention in Anthropometric Parameters

Mean body weight loss, mean visceral fat loss and mean body fat loss were the parameters used for the determination of the effect of HT in women with overweight and obesity.

The intervention and placebo groups did not have statistically significant differences at the baseline concerning the basic anthropometric parameters. Based on the study design, there were baseline differences between groups A and B; therefore, comparisons between these two groups were not performed. Comparisons of body weight and fat loss between subgroups (A and C or B and C) were made where applicable, i.e., for parameters that did not have baseline differences. Baseline anthropometric parameters are presented in Table 3 below, while Table 4, Table 5, Table 6 and Table 7 depict the variances of the measured parameters in the three time points of the intervention compared to baseline and the results of statistical comparisons.

In more detail, Table 4 presents the mean body weight loss in kilograms (kg) registered at each visit compared to the baseline. After 1 month (T1), group A, which received the high HT dosage, had an average loss of 4.31 kg, followed by groups B and C, which lost 1.22 kg and 2.45 kg, respectively. After three months of the intervention (T3), group A almost doubled the loss and lost a total of 7.97 kg on average. The same trend was observed in groups B and C, which lost 2.78 and 5.04 kg, respectively. Upon completion of the intervention after 6 months (T6), only group A experienced further weight loss, with a 10.14 kg total mean reduction of their body weight, while the remaining groups had almost stable or only slightly reduced body weight.

As the intervention was combined with lifestyle modification (diet and physical activity), participants in all study groups, including placebo, experienced body weight loss. Notably, outliers were noticed and this could explain the high standard deviation noted, especially after 6 months of the intervention. Despite the fact that the high SD could weaken the reliability of the results, the differences in body weight loss between the groups were large. When comparing group A to the placebo group, a greater mean body weight reduction was noticed in group A and was statistically significant after the first month (T1, *p* = 0.019), but statistical significance was not confirmed in the next visits (*p* = 0.096 at T3 and *p* = 0.113 at T6). Interestingly, while the dose of 15 mg HT seemed to be related to greater weight loss, this effect was not noticed in women with less severe obesity, who received the dose of 5 mg HT. Therefore, when comparing the intervention group (15 mg and 5 mg of HT) versus the placebo group, statistically significant differences did not occur.

Furthermore, a reduction of more than 5% of initial body weight was more prominent in group A compared to group C at T1 (*p* = 0.027). This superiority of group A *vs* placebo was also noted when comparing participants who lost more than 10% of initial body weight at T6 (*p* = 0.009). No statistically significant differences occurred when comparing the intervention group (groups A and B) to placebo. Table S3 in Supplementary Materials represents detailed results of these statistical comparisons.

**Table 4:** Mean body weight loss expressed in kg. Results are expressed as mean values of groups A (high dosage—15 mg HT/day), B (low dosage—5 mg HT/day) and C (placebo) at time points T1, T3 and T6. The *p*-values express the statistical difference between the intervention and placebo groups. The § marker is used for comparisons between groups A and C. SD: standard deviation.

Mean Body Weight Loss (kg)	1 Month (T1)	3 Months (T3)	6 Months (T6)
Group A	−4.31 (SD 1.83)	−7.97 (SD 4.24)	−10.14 (SD 5.41)
Group Β	−1.22 (SD 1.03)	−2.78 (SD 2.12)	−2.74 (SD 2.43)
Intervention group (groups A and B)	−2.76 (SD 2.15)	−5.52 (SD 4.25)	−6.44 (SD 5.57)
Group C (placebo)	−2.45 (SD 1.11)	−5.04 (SD 2.69)	−5.44 (SD 5.69)
*p*	0.604 § 0.019	0.714 § 0.096	0.685 § 0.113

Table 5 illustrates the mean loss of visceral fat of the three groups expressed as a percentage. In the first month of the study, the mean visceral fat loss in group A was 1.67% compared to the baseline, while groups B and C exhibited mean visceral fat mass losses of 0.22% and 0.94%, respectively. At the 3-month point of the study, the visceral fat mass was reduced by 2.67%, 0.62% and 1.71% for groups A, B and C, respectively, while only slight changes were noticed upon completion of the study. Similarly with body weight, there were no statistically significant differences between the intervention and placebo groups. Subgroup analysis revealed the superiority of group A versus placebo regarding visceral fat loss after one month of the intervention (*p* = 0.009 on T1), which was not observed in the following visits (*p* = 0.069 at T3 and *p* = 0.295 at T6). Interestingly, as it was also observed with body weight loss, the visceral fat loss in group B was less than in the placebo group. This was attributed to the study design, as participants in group B had less obesity at baseline, as shown in Table 3; therefore, it was expected for them to experience less weight and fat loss.

**Table 5:** Mean visceral fat mass loss expressed in %. Results are expressed as mean values of groups A (high dosage—15 mg HT/day), B (low dosage—5 mg HT/day) and C (placebo) for time points T1, T3 and T6. The *p*-value express statistical differences between the intervention and placebo groups. # marker is used for comparisons between groups B and C and § for comparisons between groups A and C. SD: standard deviation.

**M** **ean Visceral Fat Loss (%)**	**1 Month (** **t** **1)**	**3 Months (** **t** **3)**	**6 Months (** **t** **6)**
Group A	−1.67 (SD 1.20)	−2.67 (SD 1.44)	−3.00 (SD 2.15)
Group β	−0.22 (SD 0.44)	−0.62 (SD 0.83)	−0.56 (SD 1.14)
Intervention Group (GROUPS A and B)	−0.94 (SD 1.15)	−1.71 (SD 1.56)	−1.78 (SD 2.09)
Group C	−0.41 (SD 0.70)	−1.50 (SD 1.26)	−1.94 (SD 1.86)
*p*	0.176 § 0.009 # 0.497	0.718 § 0.069 # 0.107	0.847 § 0.295 # 0.09

Table 6 depicts the mean body fat mass loss in each visit compared to baseline, expressed in kg. After 1 month of HT administration, group A lost 3.24 kg of fat, while in groups B and C, the corresponding mean losses were 1.10 kg and 2.18 kg, respectively. In the third month, the trend of doubling was observed again, with fat mass losses of 6.29 kg, 2.68 kg and 4.17 kg for groups A, B and C, respectively. Upon completion of the study, group A continued losing body fat and participants showed a total mean fat loss of 8.16 kg. Groups B and C remained almost stable. Despite the observed differences, statistical significance was not reached between the intervention and placebo groups or between the subgroups. As was also observed with the other studied parameters, a high SD was noticed, especially in the placebo group, at the end of the intervention, where this could be attributed to the presence of outliers who followed lifestyle modification.

**Table 6:** Mean body fat loss compared to the baseline, expressed in kg. The results are expressed as mean values of groups A (high dosage—15 mg HT/day), B (low dosage—5 mg HT/day) and C (placebo) at time points T1, T3 and T6. The *p*-values express the statistical differences between the intervention and placebo groups. The § marker notes the *p*-values for comparisons between groups A and C. SD: standard deviation.

Mean Body Fat Loss (kg)	1 Month (T1)	3 Months (T3)	6 Months (T6)
Group A	−3.24 (SD 0.92)	−6.29 (SD 2.80)	−8.16 (SD 4.20)
Group Β	−1.10 (SD 0.99)	−2.68 (SD 1.85)	−2.06 (SD 1.73)
Intervention group (groups A and B)	−2.18 (SD 1.43)	−4.59 (SD 3.01)	−5.11 (SD 4.46)
Group C	−2.48 (SD 1.46)	−4.17 (SD 2.49)	−4.35 (SD 5.12)
*p*	0.586 § 0.192	0.707 § 0.094	0.711 § 0.129

Table 7 depicts the percentage variations in the abovementioned studied parameters, calculated as a percentage of the absolute loss divided by the baseline value. When comparing groups A and C, the participants that received 15 mg of HT experienced enhanced body weight visceral fat and total fat mass reduction at all study intervals compared to the placebo group, but this difference was statistically significant only for body weight and visceral fat mass at the first-month follow-up of the study.

**Table 7:** The %weight, %visceral fat and %fat mass variations of the three groups at three time intervals: T1, T3 and T6. Group A received 15 mg HT/day, group B received 5 mg HT/day and group C received a placebo. Mean values are expressed ± standard deviations. The *p*-values represent the statistical differences between the intervention and placebo groups. Statistically significant differences between groups A, B and C are reported: the # marker is used for comparisons between groups B and C and § is used for comparisons between groups A and C.

	Month 1 (T1)			Month 3 (T3)			Month 6 (T6)		
Mean % Variations ± SD	Intervention Group	Group C (Placebo)	*p*	Intervention Group	Group C (Placebo)	*p*	Intervention Group	Group C (Placebo)	*p*
%Weight	Group A: −4.82 ± 1.77 Group B: −1.67 ± 1.44	−2.98 ± 1.33	0.694 # 0.065 *§ 0.012*	Group A: −8.80 ± 4.18 Group B: −3.84 ± 2.92	−6.15 ± 3.31	0.829 # 0.170 § 0.106	Group A: −11.03 ± 5.67 Group B: −3.80 ± 3.33	−6.54 ± 6.76	0.760 # 0.325 § 0.114
%Visceral fat mass	Group A: −11.65 ± 7.30 Group B: −1.89 ± 3.78	−3.74 ± 6.06	0.245 # 0.494 *§ 0.006*	Group A: −18.21 ± 7.70 Group B: −5.58 ± 7.68	−12.25 ± 10.45	0.996 # 0.120 § 0.148	Group A: −19.16 ± 13.68 Group B: −4.75 ± 10.93	−15.82 ± 15.59	0.547 # 0.109 § 0.620
%Fat mass	Group A: −8.75 ± 2.31 Group B: −4.15 ± 3.50	−6.88 ± 4.28	0.538 # 0.066 § 0.516	Group A: −16.58 ± 6.79 Group B: −10.17 ± 6.67	−12.18 ± 7.08	0.623 # 0.535 § 0.167	Group A: −20.88 ± 11.06 Group B: −7.89 ± 6.03	−11.87 ± 13.89	0.663 # 0.470 § 0.111

Before performing the ANCOVA, a transformation of the data was made [44] and preliminary checks were completed to assess the assumptions of normality, linearity, homogeneity of the regression slopes and homogeneity of the variance. In concordance with previous results, after controlling for baseline values of each parameter, there was a statistically significant difference between groups A and C regarding the measured variation of body weight (*p* = 0.017) and visceral fat (*p* = 0.023) at T1, whereas no significant effects were noted between all other comparisons, either between groups A and C or between the intervention and placebo groups. Detailed results of the univariate analysis of covariance are depicted in Table S4 in the Supplementary Material.

Based on the above data, 15 mg of HT may be effective regarding weight and visceral fat loss as an adjunct to diet and physical activity, while the dose of 5 mg did not seem to have any favorable effects on body composition status. The indifferent findings of 5 mg of HT concerning weight and fat loss were in accordance with a previous cohort study in overweight men, which did not show any effect of 9 mg HT on weight loss [32]. In the present study, group B experienced less improvement in all the studied parameters compared to other subgroups, where this may be attributed to the fact that group B included women who were slightly overweight and, thus, who were expected to lose less body weight and fat mass compared to women with obesity. On the other hand, the encouraging findings of 15 mg of HT/day were in line with previous studies in which 15 mg of HT was administered for 3 weeks [34]. Of note, no adverse events were reported with capsule consumption.

A notable finding of our study was the attenuation of the initial encouraging effects on body composition over time, which is a common finding in similar studies. It would be expected that the initial weight loss of the first month would act as a strong motivation for the participants to comply with the capsule consumption and diet. While capsule consumption was thoroughly monitored, compliance with a diet, which is known to be difficult to achieve in the long term, was self-reported. Thus, it is possible that participants followed the diet less intensively as time lapsed, despite reporting good compliance. Of course, this effect would be expected to have an impact on both groups, but underlying differences between groups may have been undetected and, therefore, act as a confounding factor. Nevertheless, a long period of investigation is strongly advised in further relative research.

A limitation of our study is that there were baseline differences between subgroups. In order to avoid drawing misleading conclusions, statistical analysis was not performed between groups with significant baseline differences. Further research with large study populations is warranted in order to extract safe conclusions.

### 3.3. Metabolomic Analysis of Urine Samples via UPLC-Orbitrap-MS

#### 3.3.1. Samples for Metabolomics Study and Validation Aspects

In total, 40 samples were included in the metabolomic analysis coming from participants who received the HT treatment: 21 from group A (high dose) and 19 from group B (low or EFSA dose). Additionally, 23 samples came from participants who received the placebo capsule. Furthermore, QC-pooled samples were prepared from the total number of urine samples (63), which were composed of 63.5% HT samples and 36.5% placebo samples (Figure S9 in Supplementary Materials). The incorporation of QC-pooled samples is highly advisable in such studies and they are monitored throughout the analysis [45,46]. Generally, in LC-MS-based metabolomics, crucial parameters for the integrity, soundness and reliability of the generated data are the quality of samples analysis and the acquisition set-up. Thus, special attention was given to ensure the system stability, as well as to avoid any induced (technical) variability other than the biological variability, which was the objective of the study. Moreover, any undesired bias should be avoided. To this end, in the current study, the QC-pooled samples were prepared and injected in triplicate every fifty runs. The stability of the RT and peak area, as well as the mass accuracy of selected peaks, were monitored and evaluated to ensure the validity of the analysis. In the Supplementary Materials, more information about validation aspects and acquisition set-up is given, together with an overview of the urine samples that were analyzed and information about the QC-pooled (Figures S9–S11). Moreover, all samples were randomized in sequence for unbiased data processing and evaluation [45].

#### 3.3.2. Chemometrics in Urine Samples

**Figure 2:**
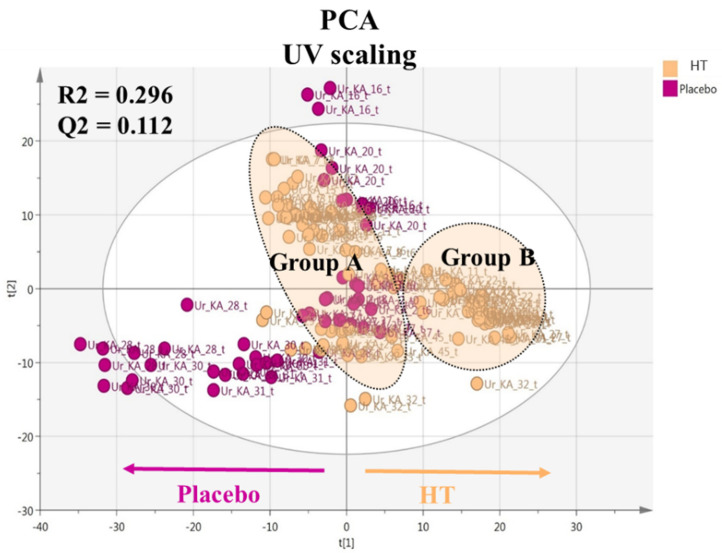
Principal component analysis (PCA) scores plot with unit variance (UV) scaling including the total number of samples. Observations (samples) are colored according to the administered capsule: purple for placebo and light pink for hydroxytyrosol (HT) capsules. Fitting parameters are also depicted.

After processing the UPLC-HRMS data, they were subjected to multivariate data analysis. Unsupervised methods and particularly PCA analysis were firstly employed to explore data fitting, the existence of possible outliers and classification trends amongst the samples. Data were treated with unit variance (UV) and Pareto methods to investigate the most appropriate scaling for data visualization. UV scaling was found to produce better fitting parameters (R2 and Q2) in the generated models and was selected for the statistical analysis. Using PCA, possible classifications amongst the samples were investigated in correlation with intervention groups (A, B and C) and weight loss. The PCA scores plot below represents the visualization of the total number of HT and placebo samples analyzed (Figure 2).

The PCA model generated nine components with poor data fitting since the R2 and Q2 values were significantly low (0.296 and 0.112, respectively). Nevertheless, a weak separation and grouping were observed between the HT and placebo groups, although a considerable dispersion was observed in both groups. Outliers were also revealed, interestingly corresponding only to three different participants and time points (KA_16_T3, KA_28_T3 and KA_30_T6). Moreover, two separate groups tended to appear between groups A and B.

**Figure 3:**
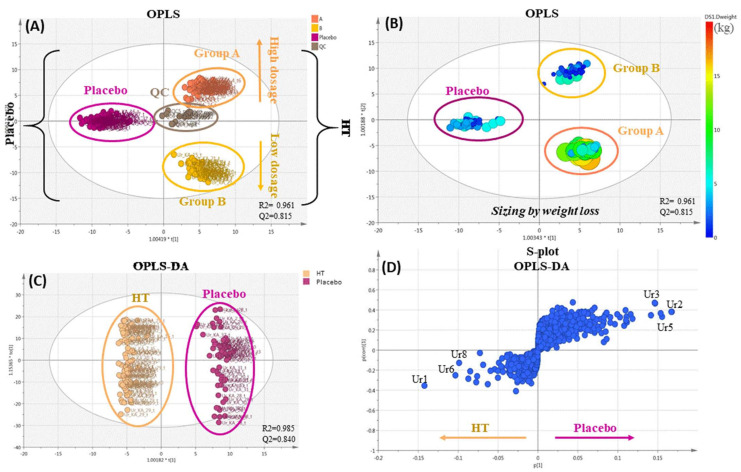
Scores and loading plots of urine samples. (**A**) Orthogonal partial least squares (OPLS) scores plot with observations colored according to the treatment group and dosage (unit variance (UV) scaling). (**B**) OPLS scores plot with observations sized and colored according to weight loss. The colored bar ranges from blue—low values to red—high values; it illustrates the weight loss in kg (UV scaling). (**C**) Orthogonal partial least squares–discriminant analysis (OPLS-DA) of the hydroxytyrosol (HT) and placebo groups (UV scaling). (**D**) S-plot of the OPLS-DA scores plot (UV scaling). The R2/Q2 model parameters are annotated (bottom-right).

Based on these initial observations, the OPLS supervised method was employed (Figure 3A). Using this method, clear clusters were revealed between the HT and placebo groups and the further subgrouping of HT into A and B groups, where high fitting parameters were determined (R2 = 0.961 and Q2 = 0.815). QC-pooled samples were also imported to investigate the model visualization efficiency. QC samples were almost centralized, revealing a discreet tendency toward the HT cluster. This observation verified the model validity given the fact that 63.5% of the QC consisted of HT samples (Figure S9 in Supplementary Material). It has to be underlined that a distinct separation was clear on the first component between the HT treatment and placebo. Additionally, separation on the second component between groups A and B was also observed.

After these initial observations of the samples classified according to intervention groups, we explored a possible correlation thereof with the weight loss observed during the study. Observations were colored and sized according to the measured weight losses at T1, T3 and T6 (Figure 3B). It is evident that the observations followed the same trend and group A presented the highest values of weight loss, while groups B and C seemed to present close values and less significant weight loss. This model is in complete accordance with the findings of Table 4, Table 5, Table 6 and Table 7, as only group A experienced statistically significant weight loss. These findings verify previous studies reporting that HT administration affects the urine metabolome of participants in a dose-dependent manner [41,47]. However, it is worth mentioning that despite the fact that in group B (low dose), significant weight loss did not occur, it seems that according to our models, the metabolome was altered in comparison to placebo and group A (high dose). Additional studies are required to further explore this issue.

OPLS-DA models were also constructed and validated through a permutation test (Figure S12) to explore the metabolites responsible for the observed clustering more in depth. Based on the loadings plot visualizing HT and placebo groups (Figure 3C), HT was detected in the samples, along with biomarkers proposed in the literature, such as homovanillic acid, HT acetate and HT sulfate [20]; however, no trend was observed according to these variables. Additionally, as is shown in the S-plot (Figure 3D), several statistically significant metabolites, based on their VIP (variable importance in projection) value, were identified, which were different for the HT (Ur1, Ur6, Ur8) and placebo (Ur2, Ur3, Ur4, Ur5, Ur7) groups (Table 8). The statistically significant metabolites of the HT group could be associated with HT administration and/or weight loss; however, further research is required.

Thus, our urine metabolomics data provided better insight into metabolic changes after HT administration and additionally underline not only the importance of the administered dose but also the duration of intake. The low-dose HT group seemed to experience metabolic changes, as reflected in their urine metabolome, though without a clinical manifestation, in contrast to the high-dose HT group. This could also be explained by the fact that group B consisted mainly of women who were only slightly overweight and who were known to have more difficulty losing weight in comparison to women with obesity. A trial of daily administration of 5 mg HT to people with higher levels of obesity could thus yield better insight. It is important to note here that the majority of available experimental studies reporting favorable effects of HT have used high doses of HT, exceeding the daily dose of 51 mg of HT, which is considered safe (0.85 mg/kg/day) [48]. Additionally, human studies should consider that HT is also present in several foods; therefore, the determination of the actual individual intake of HT may be spurious.

**Table 8:** Identified statistically significant metabolites from the orthogonal partial least squares–discriminant analysis (OPLS-DA) and S-plot. For each metabolite, a code number was given. The experimental *m*/*z* value, the suggested molecule, the molecular formula (MF), the ring and double bond equivalence (RDB eq) and the variable importance of projection value (VIP) are given. In the last column, the group of each metabolite is also noted.

	*m*/*z* Exp	Suggested Molecule	MF	RDB eq	VIP	Group
**Ur1**	178.0512	Hippuric acid	C_9_H_9_O_3_N	6.5	20.3404	HT
**Ur2**	194.0462	Hydroxyhippuric acid	C_9_H_9_O_4_N	6.5	6.30864	Placebo
**Ur3**	367.1586	Epitestosterone sulfate	C_19_H_28_O_5_S	6.5	5.59170	Placebo
**Ur4**	369.1733	5a-Dihydrotestosterone sulfate	C_19_H_30_O_5_S	5.5	5.29357	Placebo
**Ur5**	181.0505	Homovanillic acid	C_9_H_10_O_4_	5.5	5.20725	Placebo
**Ur6**	145.0616	Glutamine	C_5_H_10_O_3_N_2_	2.5	5.08479	HT
**Ur7**	261.0079	Homovanillic acid sulfate	C_9_H_10_O_7_S	5.5	4.62802	Placebo
**Ur8**	187.0073	*p*-cresol sulfate	C_7_H_8_O_4_S	4.5	4.15471	HT
**Ur9**	195.0523	1,3-Dimethyluric acid	C_7_H_8_O_3_N_4_	6.5	4.05979	Placebo
**Ur10**	245.0128	Homovanillic aldehyde sulfate	C_9_H_10_O_6_S	5.5	3.97235	Placebo

## 4. Conclusions

Overall, for the first time, a double-blinded, placebo-controlled human study was performed to investigate the anti-obesity effect of HT for a prolonged period. Based on previous studies that stated that HT persists longer in female rats and when administered in the form of OO, HT was administered to female volunteers as an encapsulated HT-enriched extract. In both capsules, refined OO was used as a carrier with the complete absence of biophenols, while HT capsules were enriched in HT that originated from edible olives’ debittering water. The intervention was safe and well-tolerated.

We found that in otherwise healthy women with overweight or obesity, the administration of 5 mg HT/day did not exert any significant effects. On the other hand, the administration of 15 mg HT/day resulted in enhanced body weight and fat reduction compared to the placebo, but this effect was statistically significant only for weight and visceral fat loss at 4 weeks into the study. UPLC-HRMS-based urine metabolomics analysis was employed, revealing that HT supplementation significantly affected the urine metabolome in a dose-dependent manner. Moreover, a significant correlation between high HT dose and weight loss was revealed, while certain urine metabolites were found to be statistically significant for each group. As the encouraging findings concerning body composition at 4 weeks of the study were attenuated over time, a long period of investigation is advised in further research. Future human studies in larger study populations may contribute further to our understanding of the metabolic effects of HT and potentially reveal significant urine biomarkers associated with weight loss.

## Figures and Tables

**Table 3:** Mean anthropometric parameter values ± standard deviation at baseline visit. *p*: statistical difference between intervention and placebo group.

Parameter	Intervention Group (*n* = 18)	Group A (*n* = 9)	Group B (*n* = 9)	Group C (*n* = 11)	*p*	Overall Mean Values (*n* = 29)
Mean ViScan value (%)	12.25 ± 3.23	13.83 ± 2.99	10.66 ± 2.76	12.73 ± 1.94	0.662	12.43 ± 2.78 (7.00 to 18.50)
Mean body weight (kg)	80.40 ± 10.92	87.60 ± 10.77	73.2 ± 4.53	82.10 ± 7.44	0.438	81.04 ± 9.63 (65.4 to 100.60)
Mean fat mass (kg)	31.98 ± 7.17	37.32 ± 5.67	26.82 ± 3.83	34.29 ± 5.76	0.373	32.85 ± 6.66 (19.1 to 45.3)

## Supplementary Materials

The following are available online at https://www.mdpi.com/article/10.3390/nu14071525/s1, Table S1: List of urine samples for metabolomic analysis, Figure S1: LC-HRMS analyses of capsules, Figure S2: HRMS full scan spectrum of hydroxytyrosol, Figure S3: HRMS full scan spectrum of dialdehydic form of decarboxymethyl elenolic acid, Figure S4: HRMS full scan spectrum of hydroxytyrosol acetate, Figure S5: HRMS full scan spectrum of octadecanedioic acid, Figure S6: HRMS full scan spectrum of linoleic acid, Figure S7: HRMS full scan spectrum of palmitic acid, Figure S8: HRMS full scan spectrum of oleic acid, Table S2: Unidentified constituents of the capsules, Table S3: Representation of the statistical differences of body weight loss among between intervention and placebo groups, Table S4: Results of univariate analysis of covariance of anthropometric parameters’ variations at T1, T3 and T6, while controlling for baseline values of each parameter, Figure S9: Representation of urine samples, Figure S10: UPLC-HRMS chromatogram of QCs-pooled, Figure S11: RSD of peaks for monitoring the validity of the analysis, Figure S12: Permutation test.
