# Supplementary material for: Effect of Long-Term Hydroxytyrosol Administration on Body Weight, Fat Mass and Urine Metabolomics: A Randomized Double-Blind Prospective Human Study
Source: Nutrients. 2022 Apr 6;14(7):1525. doi: 10.3390/nu14071525 (PMC9003406; doi:10.3390/nu14071525)
Supplement: Supplementary file 1 [file nutrients-14-01525-s001.zip › nutrients-1643096-supplementary.pdf]

SUPPLEMENTARY MATERIAL

Effect of long-term hydroxytyrosol administration on body weight, fat mass and urine metabolomics. A randomized double-blind prospective human study.

Christina Fytili[1#], Theodora Nikou[2#], Nikolaos Tentolouris[1], Ioulia K. Tseti[3], Charilaos Dimosthenopoulos [4], Petros P. Sfikakis [1], Dimitrios Simos [1], Alexandros Kokkinos[1], Alexios L. Skaltsounis[2], Nikolaos Katsilambros[4], Maria Halabalaki[2*]

1. Urine samples for metabolomic analysis

Table S1. List of urine samples for metabolomic analysis. List of urine samples included in metabolomic analysis via Ultra-Performance Liquid Chromatography–High-Resolution Mass Spectrometry (UPLC-HRMS). The first column represents the given code to the participants and T0, T3 and T6 the sample collection at the baseline visit, the third and the sixth month, respectively. The last column illustrates participants group.

Patient code	T0	T3	T6	Group
KA_2	✓	✓	✓	C
KA_3	✓	-	-	C
KA_4	✓	✓	✓	A
KA_7	✓	✓	✓	A
KA_8	✓	✓	✓	B
KA 9	✓	✓	✓	B
KA_10	✓	-	-	A
KA_11	✓	✓	✓	A
KA_14	✓	✓	✓	C
KA_15	✓	✓	✓	B
KA_16	✓	✓	-	C
KA_20	✓	✓	✓	C
KA_21	✓	✓	✓	B
KA_22	✓	✓	✓	A
KA_24	✓	✓	✓	A
KA_27	✓	✓	-	B
KA_28	✓	✓	✓	C
KA_29	✓	✓	✓	A
KA_30	✓	✓	✓	C
KA_31	✓	-	✓	C

KA_32	✓	✓	-	B
KA_33	✓	✓	-	A
KA_37	✓	✓	✓	C
KA_45	✓	✓	✓	B
**Sum**				
**Urine**	**63 samples**			

2. Qualitative and quantitative analysis of capsules

For the quantitative analysis of capsules (HT and placebo), a Supelco Discovery HS C18 (25 cm x 4.6 mm, 5 μm) column was used. The elution gradient was consisted of H_2O + 0.2% acetic acid (solvent A) and ACN (solvent B). The separation started with 2% of B and in seventeen minutes reached 30% of B. Finally, after three minutes the system returned to the initial conditions. The flow rate was 1 mL/min and the injection volume was 20 μL. The total acquisition time was 20 minutes and the quantitation and monitoring took place at λ=280 nm (λmax of HT). Capsules treatment and HPLC-DAD analysis were performed in triplicate. Spectra recording and data processing were carried out with ChromQuest™ 4.1 software (Thermo Scientific, Massachusetts, USA).

For the qualitative analysis of capsules, a UPLC-HRMS method was employed. For the separation, H_2O with 0.1% formic acid was used as solvent A and ACN as solvent B. The elution method started with 2% of B and stayed in these conditions for two minutes. The next sixteen minutes the percentage of B increased to 100% and maintained for three minutes. Finally, at twenty-ninth minute, A reached the initial conditions and stayed for 4 minutes for system equilibration. An Acquity UPLC Peptide BEH C18 (100 mm x 2.1 mm, 1.7 μm) column was used for the analysis with stable temperature at 40°C. The measurements were performed with a total acquisition time of 25 minutes and a flow rate of 400 μL/min. The injection volume was 10 μL and the autosampler temperature was at 7°C. Mass spectra were obtained in negative and positive ionization. For the negative ionization the capillary temperature was set at 350°C, capillary voltage at -30 V and tube lens at -100 V. Sheath and auxiliary gas were adjusted at 40 and 10 arb, respectively. For the positive ionization the above parameters were retained and only capillary voltage and tube lens were adjusted to 40V and 120 V, respectively.

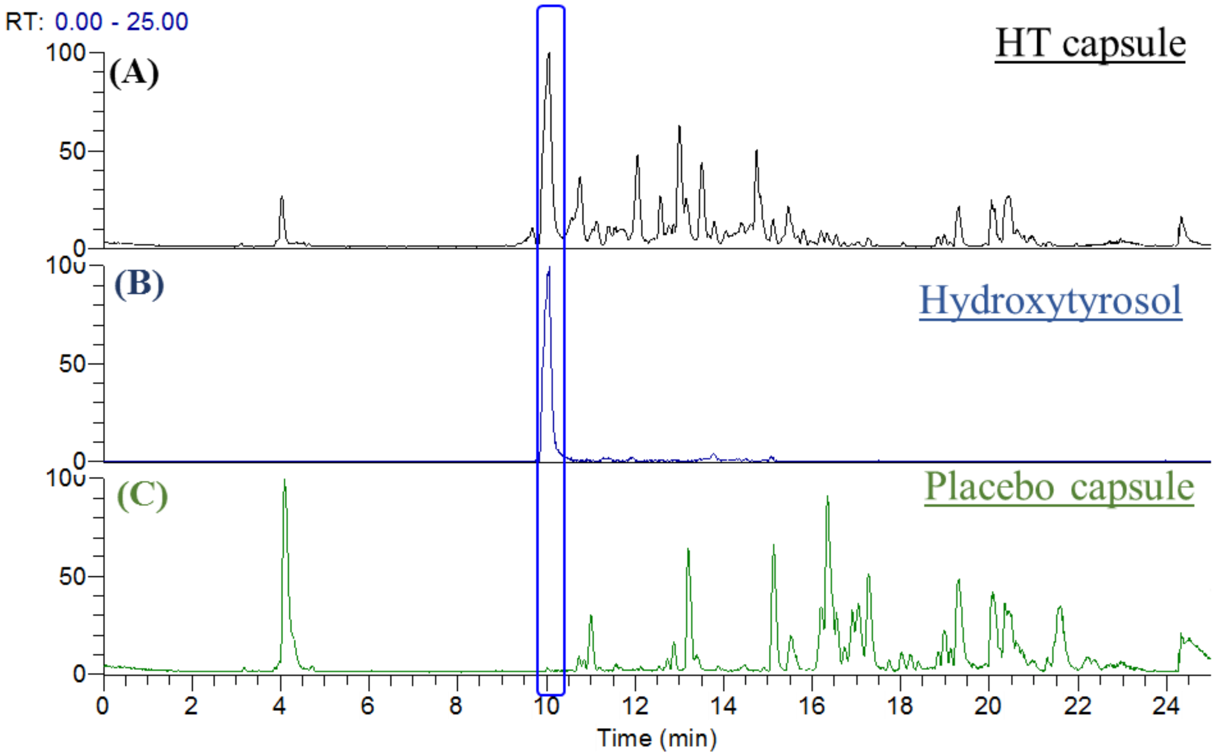

Figure S1. *LC-HRMS analyses of capsules. Liquid Chromatography–High-Resolution Mass Spectrometry(LC-HRMS) base peak (BP) chromatogram of hydroxytyrosol (HT) capsule (A), reference standard of HT (B) and base peak (BP) chromatogram of placebo capsule (C) analyzed in negative ionization (ESI*$^-$*). HT is circled in blue. RT: retention time.*

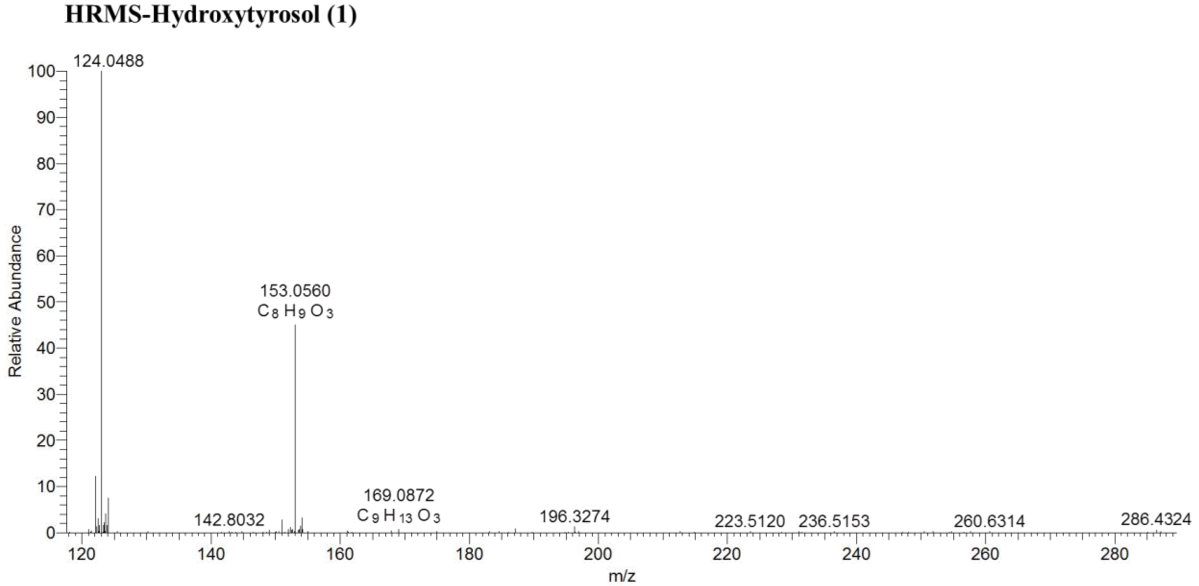

Figure S2. *HRMS full scan spectrum of hydroxytyrosol (**1**)*

HRMS-Dialdehydic form of decarboxymethyl elenolic acid (2)

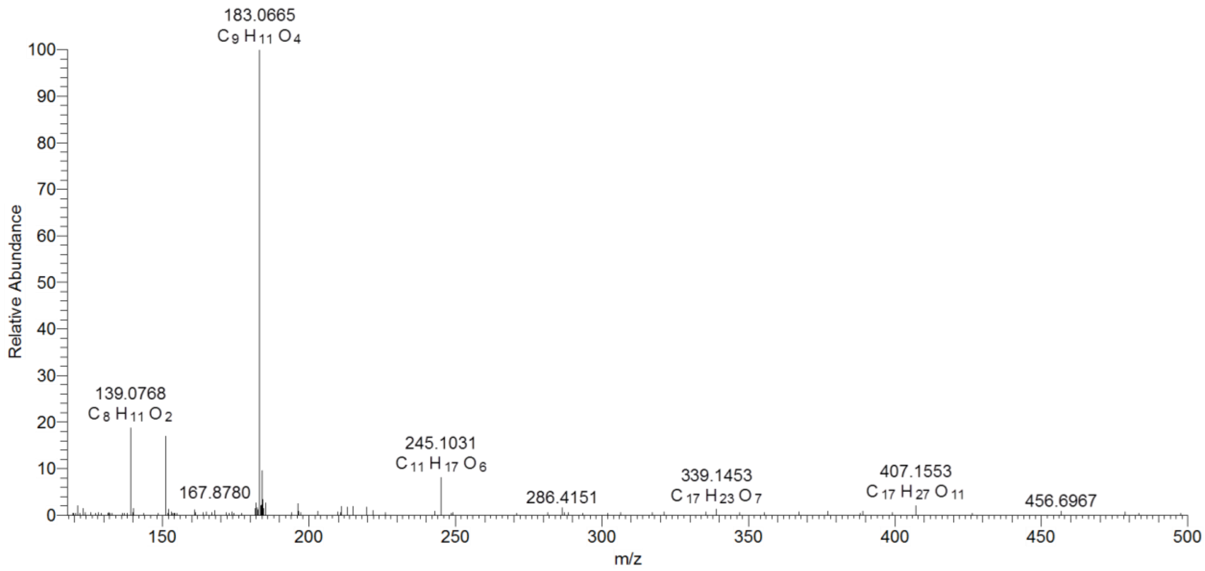

Figure S3. *HRMS full scan spectrum of dialdehydic form of decarboxymethyl elenolic acid (**2**)*

HRMS-Hydroxytyrosol acetate (3)

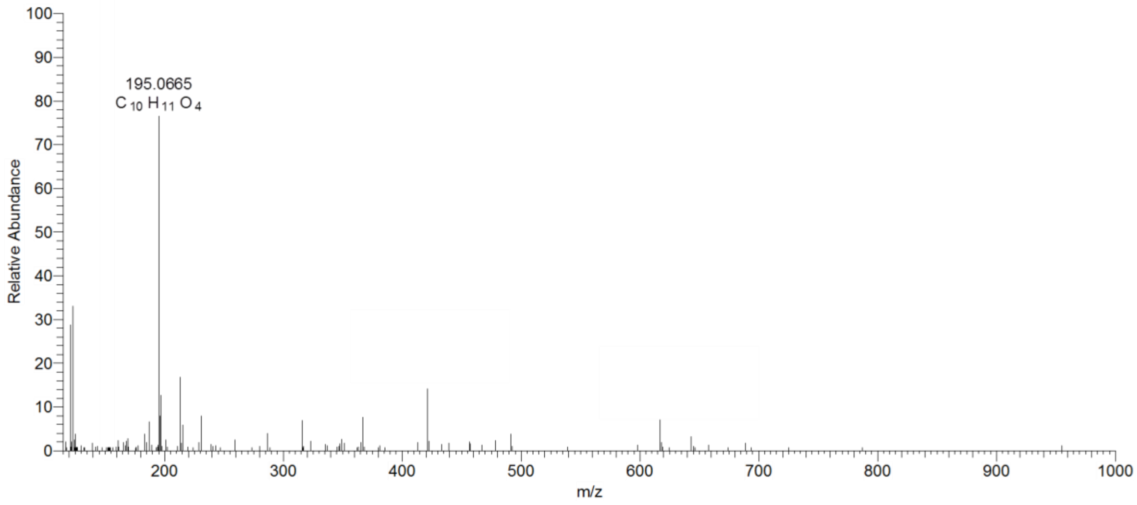

Figure S4. *HRMS full scan spectrum of hydroxytyrosol acetate (**3**)*

HRMS-Octadecanedioic acid (4)

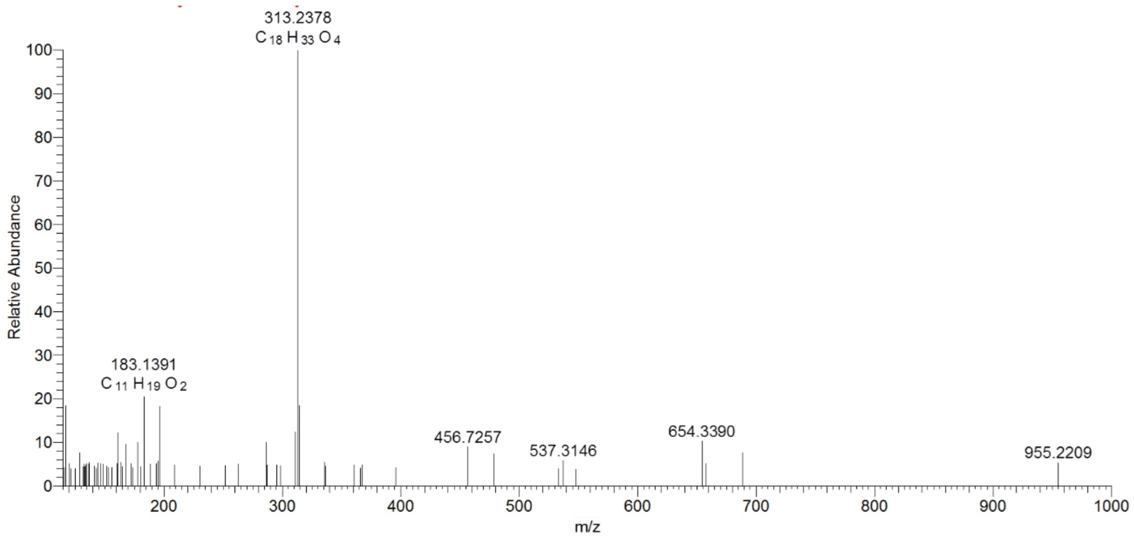

Figure S5. *HRMS full scan spectrum of octadecanedioic acid (**4**)*

HRMS-Linoleic acid (5)

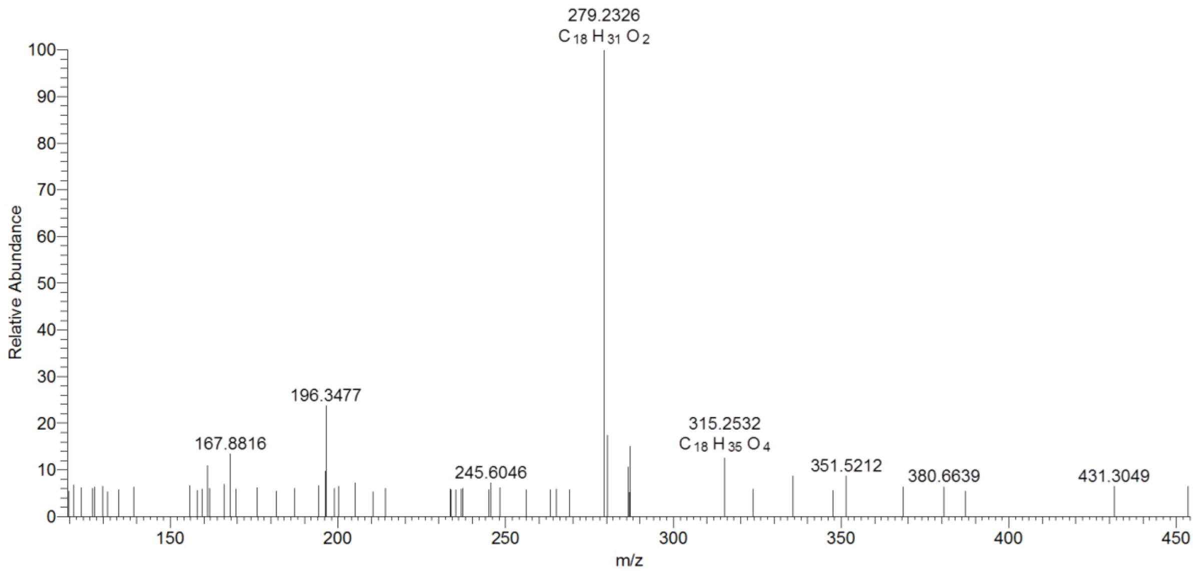

Figure S6. *HRMS full scan spectrum of linoleic acid (**5**)*

HRMS-Palmitic acid (6)

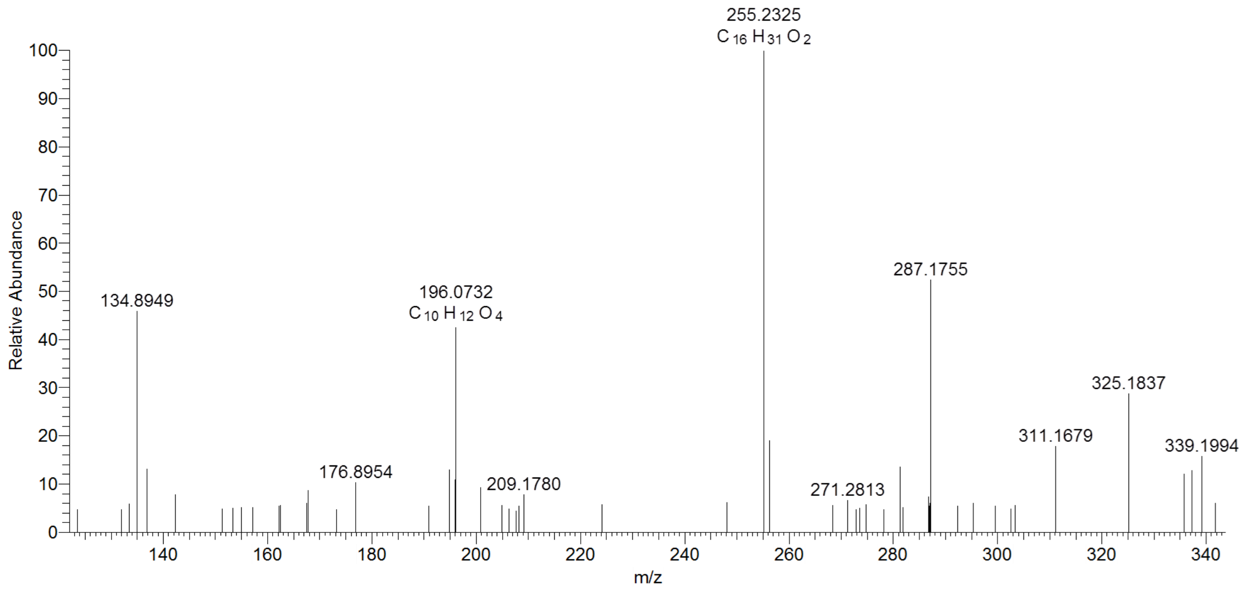

Figure S7. *HRMS full scan spectrum of palmitic acid (**6**)*

HRMS-Oleic acid (7)

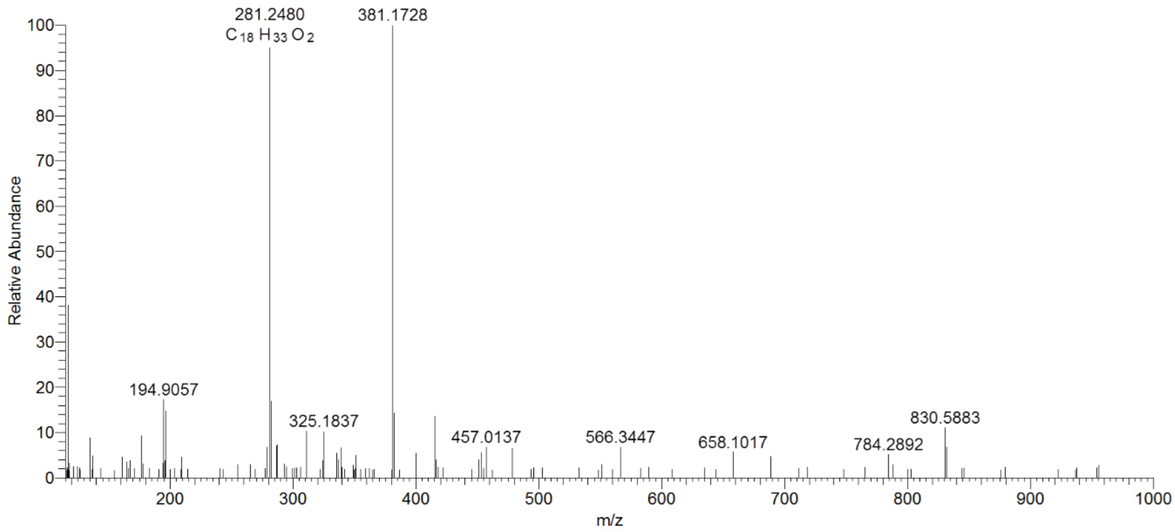

Figure S8: *HRMS full scan spectrum of oleic acid (**7**)*

Table S2. Unidentified constituents of the capsules. RT: retention time and *m/z* exp: experimental *m/z* are included.

RT (min)	*m/z* exp
4.03	341.1083
9.69	407.1552
10.76	151.0768
11.42	151.0767
12.56	243.0874
13.00	197.0820
13.51	349.1290
13.78	321.1339
14.75	329.2331
15.10	331.2884
15.47	197.0819

3. Effect of the intervention in anthropometric parameters

***Table S3**. Representation of the statistical differences of body weight loss between intervention and placebo groups.*

Statistical difference among groups	Body weight loss > 5%	Body weight loss > 10%
**1 month (T1)**	p=0.472<br>§0.027	-
**3 months (T3)**	P= 0.472<br>§0.808	p=0.346<br>§0.089
**6 months (T6)**	p=0.561<br>§0.554	p=0.137<br>§0.009

§marker is used for comparisons between groups A and C.

N/A : not applicable, as none of the participants experienced weight loss more than 10% of their initial weight after 1 month of the intervention

Table S4. *Results of univariate analysis of covariance of anthropometric parameters' variations at T1, T3 and T6, while controlling for baseline values of each parameter. p= statistical difference. Statistically significant differences (<0.05) are marked in italics.*

		**p between intervention and placebo group**	**p between groups A and C**
**Mean weight loss**	T1	0.436	*0.017*
	T3	0.615	0.379
	T6	0.987	0.643
**Mean visceral fat loss**	T1	0.183	*0.023*
	T3	0.701	0.173
	T6	0.933	0.957
**Mean fat mass loss**	T1	0.696	0.150
	T3	0.584	0.361
	T6	0.533	0.601

4. Validation aspects of metabolomic analysis

In LC-MS-based metabolomics a crucial parameter for the integrity, soundness and reliability of the generated data is the repeatability of the method which is used and generally the quality of the acquisition as well as the stability of the system. Potential instability in chromatographic and MS performance as well as ionization problems (signal suppression) might affect significantly the generated data. Therefore, the repeatability and reproducibility of the acquisition should be ensured. To that end, randomization of samples and monitoring of the consistency of RT, peak area and *m/z* measurement accuracy should be monitored throughout the acquisition. For this reason, quality control (QC) pooled samples which comprise the mixture of all analysed samples are prepared, analysed and monitored [1, 2].

In the current study a QC-pooled sample was prepared and injected in triplicate every fifty runs. In figure S9 on overview of urine samples which were analysed and information about the QC-pooled sample is given

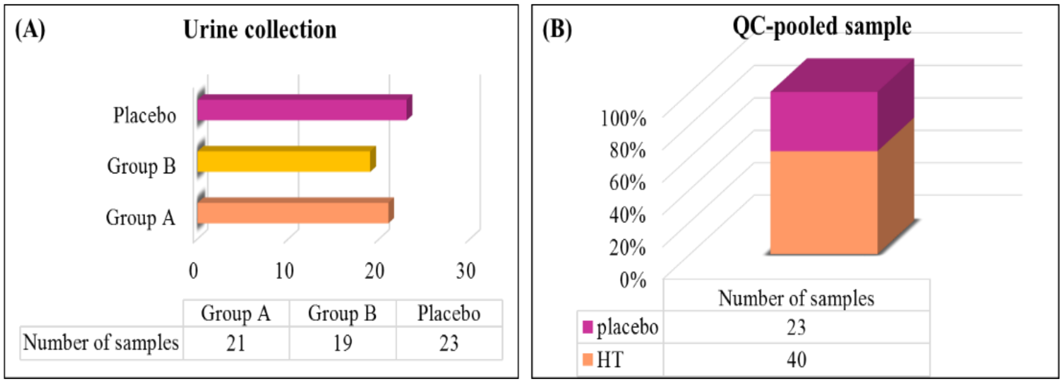

Figure S9. *Representation of urine samples. Graph* ***(A)*** *illustrates the collected samples per group; placebo samples (purple bar), group B (yellow bar) and group A (light pink bar). Graph* ***(B)*** *illustrates the composition of quality control (QC)-pooled sample; purple part of the bar represents placebo samples and light pink the hydroxytyrosol (HT) samples.*

As mentioned above, in order to ensure the metabolomic analysis validity, three peaks from the QC-pooled sample were selected and the parameters of RT, mass accuracy and peak area were monitored throughout the sequence in each QC run. The selection of the peaks was based in order to cover different 1) RT, 2) mass range and 3) peak area level. More specifically, hippuric acid (1) (*m/z*: 178.0512, RT=4.82 min), cortolone-3-glucuronide (2) (*m/z*: 571.2665, RT=7.59 min) and laurylsulfate (3) (*m/z*: 265.1479, RT=9.6 min) were selected as the peaks satisfy RT, mass range and peak area window. Figure S10 illustrates a QC injection. The three selected compounds are annotated.

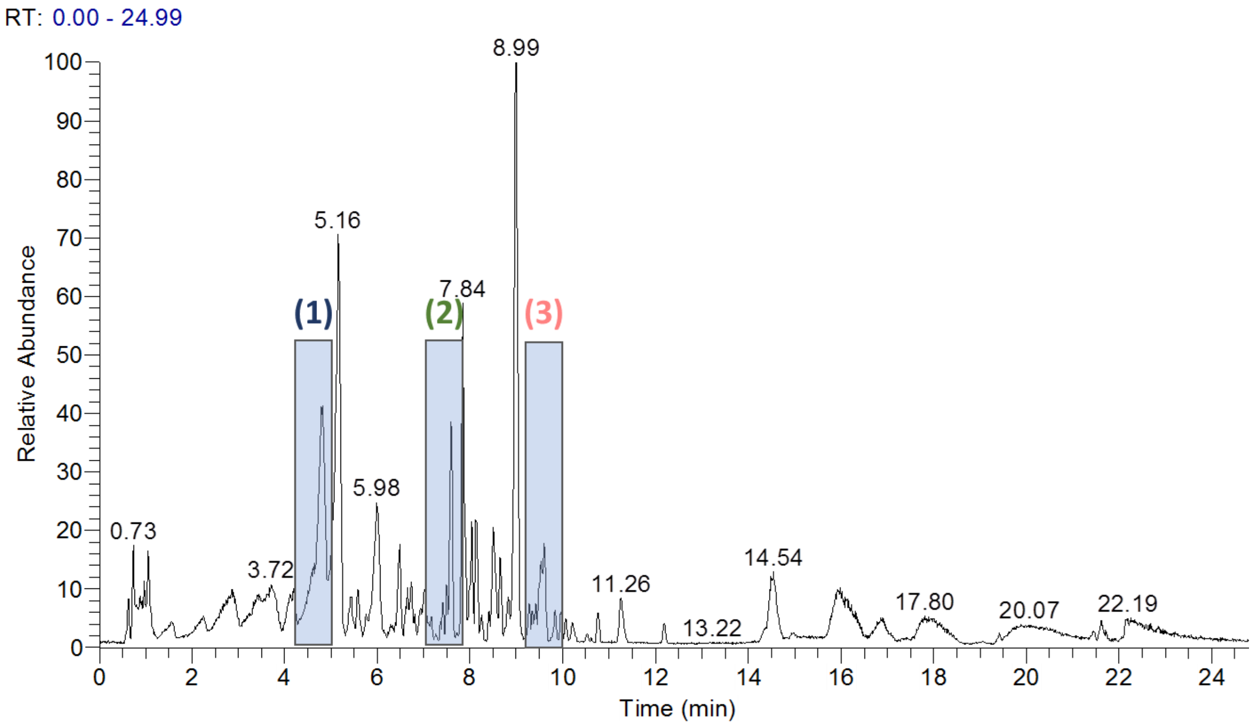

Figure S10. *UPLC-HRMS chromatogram of QCs-pooled. Ultra Performance Liquid Chromatography–High-Resolution Mass Spectrometry (UPLC-HRMS) base peak chromatogram of QC-pooled sample in negative ionization mode. Annotated peaks* ***1****,* ***2*** *and* ***3*** *represent the selected compounds used for the evaluation of the repeatability of the analysis. RT: retention time.*

After the selection of the peaks, %RSD for RT and peak area were evaluated and acceptance thresholds were set based on FDA guidelines [2]. In more detail, % RSD should be less than 1% for RT and 10% for peak area. Also, accuracy should be less than 5 ppm in all QC-pooled measurements. The figure below illustrates the %RSD of RT for the three selected metabolites hippuric acid, cortolone-3-glucuronide and laurylsulfate.

As it is shown in figure S11, % RSD values of the three metabolites were found 0.34% for hippuric acid, 0.20% for cortolone-3-glucuronide and 0.21% for laurylsulfate. The same estimations were performed for area parameter. In brief, the calculations showed %RSD for RT<0.4%, %RSD for area <8.2% and accuracy<2 ppm. Therefore, the analysis was accepted as accurate and repeatable for samples analysis.

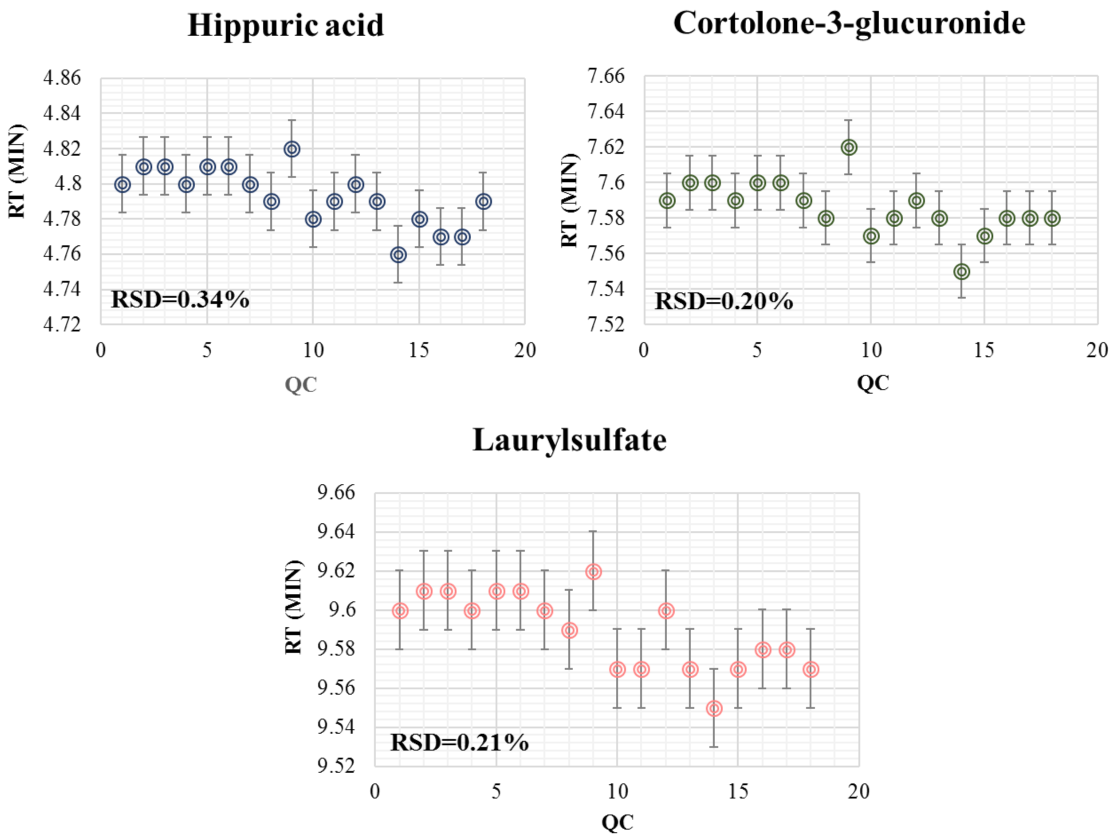

***Figure S11**. RSD of peaks for monitoring the validity of the analysis. Representation of relative standard deviation (RSD),%) for retention time (RT) of hippuric acid (blue symbols), cortolone-3-glucuronide (green symbols) and laurylsulfate (pink symbols) in the different quality control (QC) injections within the entire sequence. The round symbols represent the mean values for RT and the bars the respective standard deviation (SD) of the values.*

The final acquisition of samples included 263 injections. In more detail, the acquisition started with 5 blank samples (MeOH) for the conditioning of the system followed by a triplicate of a standard compound (rutin) in triplicate, used as analytical QC and a triplicate of QC-pooled sample. Then, the analysis of samples started which run in triplicate and every five runs a blank sample was entered. QC samples along with rutin run also twice in triplicate during the acquisition and at the finalization.

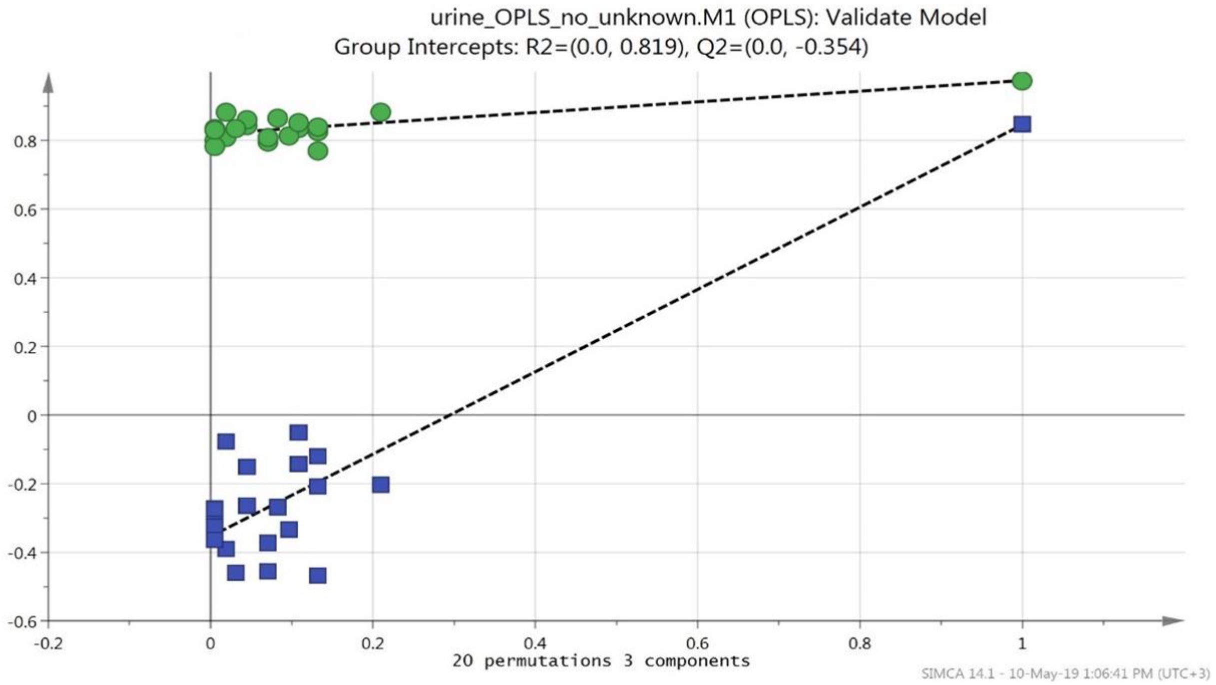

Figure S12. *Permutation test. Results of permutation test describing R2 and Q2 intercepts for all the Y variables (group A, group B, placebo and QC) of figure 3A.*

Bibliography

1. Naz, S.; Vallejo, M.; García, A.; Barbas, C. Method validation strategies involved in non-targeted metabolomics. *J. Chromatogr. A* **2014**, *1353*, 99–105, doi:10.1016/j.chroma.2014.04.071.
2. Food and Drug Administration, D. of H. and H.S. Bioanalytical Method Validation Guidance for Industry. **2013**.
